# Ring Avulsion Injuries

**Published:** 2016-02-10

**Authors:** Matt Jones, Sameer Gujral

**Affiliations:** Department of Plastic Surgery, Royal Devon & Exeter Hospital, Exeter, Devon, England

**Keywords:** ring, avulsion, amputation, reimplantation, finger

**Figure:**
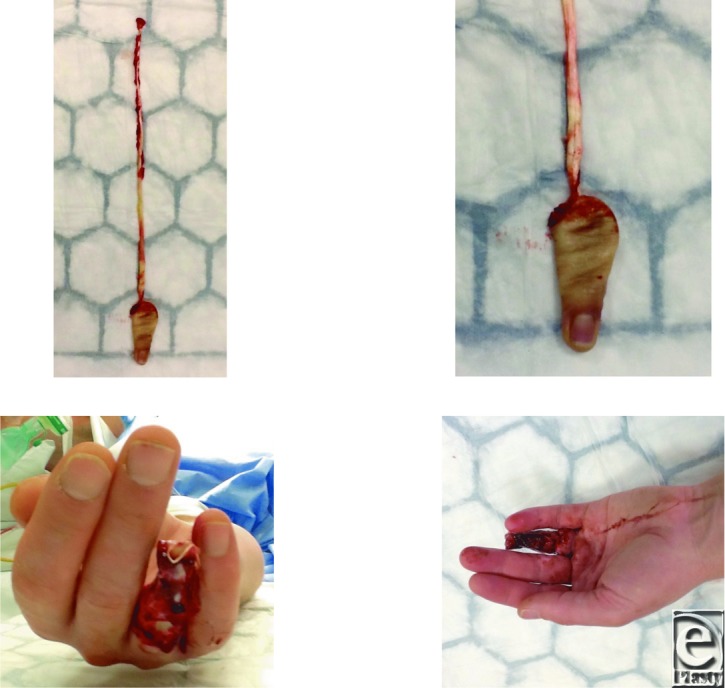


## DESCRIPTION

This right hand–dominant teacher sustained a left ring finger avulsion injury, including avulsion of his flexor digitorum profundus tendon, catching his wedding ring climbing down from a fence. There was no fracture. Flexor digitorum superficialis was intact. The digit terminalized at the level of the mid-proximal phalanx.

## QUESTIONS

1. **What is a ring avulsion injury?**
2. **How are ring avulsion injuries classified?**
3. **What are the different management options for ring avulsion injuries?**
4. **What are the long-term functional outcomes and prognosis of ring avulsion injuries?**

## DISCUSSION

A ring avulsion injury is sustained by a sudden force pulling a ring from a finger. This can result in severe injury ranging from circumferential soft-tissue laceration to complete amputation. It may involve crushing, shearing and avulsion of neurovascular bundles along with additional flexor tendon, and boney injuries. It usually involves only 1 digit and classically occurs when a wedding band is caught on machinery or a protruding object.

Urbaniak et al^1^ classified these injuries into 3 classes based on the circulatory status: class I—circulation adequate; class II—circulation inadequate; and class III—complete degloving or complete amputation. Nissenbaum^2^ subsequently modified class II (circulation inadequate) adding class IIA for isolated arterial injuries. Kay et al^3^ described an alternative classification emphasizing the presence of skeletal injury, reflecting the difference in treatment required and predicted functional outcome. In cases where there was inadequate circulation, they were divided into the absence of (class II) or presence of (class III) fracture or joint injury with only inadequate arterial (a) or venous (v) circulation. Circulation adequate injuries were still considered class I and complete amputations or gloving as class IV (Table 1).

As for any hand injury, appropriate first aid is given, and the patient is stabilized and given adequate analgesia. The amputated part, if completely avulsed, is preserved appropriately and the wounds assessed. Radiographs should be taken of the injured digit and the amputated part. Admission for elevation, analgesia, and intravenous antibiotics should be initiated, and the patient counseled for appropriate urgent surgical management. Urbaniak suggested management of injuries based on their class. Class I injuries require repair of soft tissues and fixation of any bone injury. Class II injuries (inadequate circulation) require urgent operative assessment and possible revascularization. Urbaniak et al^1^ supported revision amputation for class III injury (complete degloving/amputation) due to poor functional outcome. Avulsion amputation poses particular problems due to long-segment neurovascular bundle injury involving crush and shearing forces. Microsurgery has allowed for revascularization and replantation, along with local flap, pedicle flap, and graft coverage for further reconstruction. More recent literature also recommends attempting replantation in complete finger avulsion injuries with a preserved proximal interphalangeal (PIP) joint and flexor digitorum superficialis tendon insertion.^4^

Urbaniak class I avulsion injuries (ie, adequate circulation) have good reported functional outcomes, with only slight reduction in PIP) joint motion; one study quoting an average of 94.4° (normal = 100°).^5^ Outcomes for Urbaniak class II injuries (ie, avulsion with inadequate circulation) depend on the structures involved. Average PIP joint motion of 88.3° has been reported. PIP joint motion is worse if the flexor tendon is concomitantly injured and more so if there is a fracture. Patients with class II injuries have been shown to preserve or regain protective sensation: 7- to 10-mm static 2-point discrimination.^5^ In Urbaniak class III injury (complete avulsion), mean survival for reimplantation is 78% compared with 80% to 90% in finger reimplantation following amputation in general. An average total active motion, following replantation for class III injury, has been found to be 177° versus 199° in class II injuries. A normal range is 260° to 270°. For comparison, average recovered total active motion after isolated zone II flexor tendon repair is reported as 229°. The mean 2-point discrimination in patients after class III injury is 10 mm.^6^

Ring avulsion represents a spectrum of soft tissue, neurovascular, tendon, and bone injury. The most severe forms of injury involve vascular compromise or complete amputation, associated with tendon injury and/or fracture. Optimal management will vary depending on the pattern of injury, status of the amputated part, and functional requirements and expectation of individual patients. Classification systems are useful to stratify an approach to recommended treatment and to predict possible outcomes, which is of value when counseling patients preoperatively.

## Figures and Tables

**Table 1:** Ringer finger avulsion classifications

Urbaniak classification^1^
I Avulsion injury with adequate circulation
II Avulsion injury with inadequate circulation
III Complete degloving or complete amputation
Nissenbaum modification of Urbaniak classification^2^
IIa Avulsion injury with inadequate circulation (only arteries injured)
Kay classification^3^
I Adequate circulation, with or without skeletal injury
II Inadequate circulation (arterial and venous), no skeletal injury
III Inadequate circulation (arterial and venous), fracture of joint injury present
IV Complete amputation
a. Arterial circulation inadequate only
b. Venous circulation inadequate only
